# Biological Extremity Reconstruction after Sarcoma Resection: Past, Present, and Future

**DOI:** 10.1155/2013/529349

**Published:** 2013-06-06

**Authors:** Lukas A. Holzer, Andreas Leithner

**Affiliations:** Department of Orthopaedic Surgery, Medical University of Graz, Auenbruggerplatz 5, 8036 Graz, Austria

## Abstract

In sarcoma surgery besides a wide local resection, limb salvage became more and more important. Reconstruction of bone and soft tissue defects after sarcoma resection poses a major challenge for surgeons. Nowadays a broad range of reconstructive methods exist to deal with bony defects. Among these are prostheses, bone autografts, or bone allografts. Furthermore a variety of plastic reconstructive techniques exist that allow soft tissue reconstruction or coverage after sarcoma resection. Here we discuss the historical highlights, the present role, and possible future options for biological reconstruction.

## 1. Introduction

**Figure 1:**
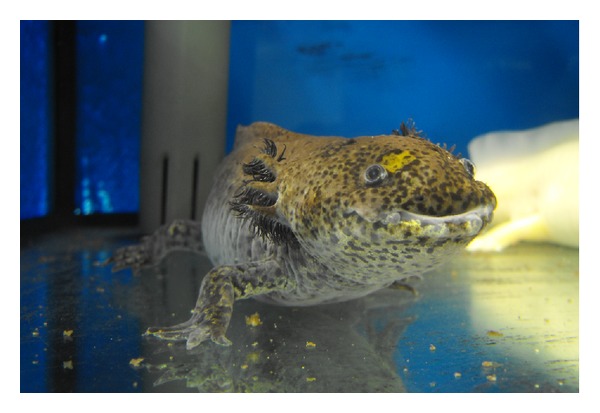
Axolotl or Mexican salamander (*Ambystoma mexicanum*), one of the few species that maintains the ability to regenerate its tail or limbs throughout its life. Karol Głąb/Wikipedia Creative Commons.

Bone and soft tissue defects after sarcoma resection pose a major challenge for surgeons [1]. While in animal life the regeneration of whole extremities is possible in some species such as starfish [2] or salamanders (Figure 1) [3, 4], in humans surgeons are dependent on advancement and innovations in biological and mechanical reconstruction. In sarcoma surgery besides a wide local resection, limb salvage became more and more important [5]. Mechanical stability and strength and the possibility of reattachment of tendons and ligaments are necessary to achieve motion and good function in reconstructed limbs. Various methods and techniques have been developed and optimized that allow maintaining the function of the extremity. To achieve this, bony defects can now be reconstructed with prostheses, bone autografts, or bone allografts. Furthermore a variety of plastic reconstructive techniques exist that allow soft tissue reconstruction or coverage. Additionally different factors are studied that might promote graft incorporation and bone repair. The aim of this review is to give an overview of historical highlights, the present role of bone grafts, and possible future options for biological reconstruction. 

## 2. Past 

The first evidence of human bone allografts in medical literature can be found in a paper published by MacEwen in 1887 [6]. The outcome of this method used in four nononcological cases was published in *Annals of Surgery* in 1909 [7]. One of the first cases which was treated in 1874 was a three-year-old boy with an osteomyelitis of the right humerus. The necrotic bone was removed surgically resulting in a deformed and useless humerus after a fifteen-month of followup. The boy's parents wanted MacEwan to have the boy's arm amputated. MacEwen however performed a bone allograft transplantation to reconstruct the humerus in three stages over a period of five months.

Erich Lexer, a German surgeon, treated septic arthritis and osteomyelitis by the use of long-bone transplants [8]. Lexer used bone allografts from fresh amputated limbs. A success rate of 50% was reported regarding the outcome of normal limb function following half-joint and whole joint osteoarticular transplantations [9]. 

Often there had been a shortage of bone allografts as grafts were obtained from amputees in most cases [10]. Furthermore there was a lack in the capacities for long-term storage and preservation [10]. Therefore autografts were preferred in many surgical situations [10]. A few decades later, two events revolutionized the situations for the use of bone allografts: on one hand, the establishment of bone or tissue banks. The first one was the Navy Tissue Bank in Bethesda, Maryland, which was established in 1949 and provided the optimal conditions for storing bone or other tissue and preserve it for a later use [11]. The second event was the invention of the lyophilization (freeze-drying) of bone [12]. As a result the management of bone allografts became more simple. Subsequently the use of bone allograft transplantation gained more and more popularity.

Parrish was the first who used the long-bone transplantation techniques described by Lexer for limb salvage in the proximal femur and distal femur for primary high-grade malignancies in bone [13]. This work was continued by other surgeons including Enneking and Mankin. Enneking et al. showed that a good incorporation of allografts is difficult to achieve due to factors such as infection or fatigue fractures [14]. Mankin et al. showed that about 70% of patients treated with long-bone allografts achieved good clinical results [15]. It was further demonstrated that incorporation of allografts might be negatively influenced by the use of radiation or chemotherapy [16, 17].

In the late 1980s, Capanna et al. introduced an interesting concept of hybrid reconstruction combining allograft shell and free vascularized fibula for large defects after tumor resection. Their hybrid graft offers initial stability and the option of reattachment of tendon and ligaments given by the allograft as well as good biological incorporation due to the vascularized fibula. Additionally soft tissue coverage can be achieved in cases of composite flaps [18]. 

The first case of limb salvage by the use of a homologous limb transplant can be found in the *legenda aurea *[19]. According to the legend the twin saints Cosmas and Damian performed an amputation in the deacon Justinian due to “cancer” [19]. The importance of this legend, however, results from the subsequent treatment. The leg was reconstructed with a homologous limb transplant. The donor was an Ethiopian who had died some hours before. The legend also provides the outcome of the deacon who was able to walk again and glorify his doctors.

Carl Nicoladoni, an Austrian surgeon, made remarkable contributions to surgery. Among those were the first thumb reconstructions. In 1897, he published three cases of thumb reconstruction using autografts from the chest. Furthermore he proposed the concept of toe-to-thumb transfer for thumb reconstruction in the same paper [20].

The first hand transplantation has been performed in 1964 in Ecuador however unsuccessfully resulting in an amputation. As immunological understanding advanced, the first successful hand transplantation has been performed in the late 1990s. Since then about 70 hand transplantations have been performed worldwide [21].

## 3. Present

### 3.1. Bone Banks

The advancements of bone banks are obvious. Meanwhile well-organized bone or tissue banks exist worldwide that provide bone allografts of different size, shape, and quantity to suit the need of surgical reconstruction [22]. This is furthermore reflected by their use in clinical practice. Procedures with allograft increased 14-fold between 1985 and 1996 in the United States of America and account for approximately one third of bone grafts today [23]. Allografts are the most commonly used bone substitutes in Europe [24]. However with the increasing number of bone allografts used there is also an increased demand for supply. Living donors, multiorgan donors, or postmortem donors are the source for bone allografts [24]. Recently a novel concept was introduced. Allografts from a bone bank are scanned using CT and reconstructed three-dimensionally. Preoperatively the most appropriate graft can be selected that matches the host's anatomy and surgical defect best [25].

### 3.2. Types of Allografts

Various types of bone allografts ranging from small cancellous bone allografts to large vascularized bone grafts are used to reconstruct bony defects [24]. These include the following ones.

Corticocancellous bone allografts are prepared from femoral heads or from long bones of the extremities. These grafts have osteoconductive property only and provide some mechanical support depending on their preparation. Corticocancellous bone allografts are widely used [24].

Demineralized bone matrix is the only bone allograft with osteoinductive capacity. These allografts contain bone morphogenetic proteins and collagen type I which are needed for the osteoinduction to occur. Various types of such allografts are available among them calcium sulphate or porcine collagen enriched demineralized bone matrices. Their use is becoming increasingly popular especially in the treatment of delayed fracture healing or nonunions [24].

Massive structural bone allografts are primarily used for limb salvage procedures in musculoskeletal oncology and pose an option for the anatomical reconstruction of large skeletal defects supporting concomitant surgical interventions such as prosthesis, osteosynthesis, or vascularized bone graft. Among the forms of structural bone allografts there are osteochondral allografts, intercalary allografts, and segmental allografts with arthrodesis or prosthesis or cortical struts. Furthermore osteoarticular allografts are available, for example, for the reconstruction after resection of the proximal humerus or of the distal radius for tumors [26, 27].

### 3.3. Complications

The most common and devastating complications in bone allograft use are nonunions with an incidence of about 10% to 25% [28, 29], fractures with an incidence of up 20% [30], and infections. High infection rates can be seen in allograft use ranging from about 20% to 70% [31]. The avitality of these grafts is believed as a cause for the high infection rates. High rates of infection are seen in areas that are poorly vascularized such as the pelvis in up to half of the recipients, whereas infection rates in extremities are much lower (5%) [29]. In general, the pelvic region is critical with about half of the reconstructions resulting in failure [29]. Other major complications include degeneration in osteoarticular grafts and epiphyseal slip in younger patients [29].

### 3.4. Safety Issues

An important issue in bone allograft transplantation is safety concerning disease transmission. Just three years after the first reported cases of AIDS the first HIV-1 transmission in bone occurred in 1984 [32]. Furthermore a few cases of hepatitis C virus infections were reported resulting from the use of bone transplantations [33, 34]. Therefore the safety of bone transplantation gained more attention, and methods of screening donors changed and improved over time. Nowadays more sensitive serologic tests are available for HIV antibodies. HIV antigens and polymerase chain reaction (PCR) are available for screening. Additionally donors' history is checked for risk factors. Furthermore it had been shown that the removal of blood and bone marrow is beneficial and reduces risk of disease transmission [35]. The estimated risk to obtain an allograft from an unrecognized HIV-infected donor is one in 1.6 millions [36].

Another topic that is under discussion currently is the antigenicity of donor material. In general, bone allografts show a low antigenic nature. This fact can be attributed mainly to the preparation and preservation of the allografts. Freeze-dried cortical bone allografts failed to sensitize recipients [37]. Still on the long term, collagen and matrix of allografts can lead to an immune response. Bone graft immunogenicity is mainly attributed to human lymphocyte antigens (HLA) that are controlled by the major histocompatibility complex (MHC) in humans. These antigens are expressed on the cell surface and represent the primary stimulus for transplant tissue rejection when HLA mismatches occur between donors and recipients. Detection of donor-specific anti-HLA antibody formation in a patient receiving bone allografts is an important measure of the clinical immunogenicity of the respective graft material [38]. A chronic type of rejection or an immunologic state of tolerance can occur in bone allograft recipients. Immunologic reaction between host and allograft has an effect on graft incorporation. New bone formation might be reduced and revascularization is delayed or even inhibited [39].

### 3.5. Types of Autografts

Autogenous bone grafts are used commonly to reconstruct bone voids or to induce bone healing. Cancellous autografts provide good osteoconductive, osteoinductive, and osteogenic characteristics, whereas cortical autografts provide osteoconductive features mainly. The most popular site for autogenous bone grafting is the iliac crest. Alternative sites include the proximal tibia, the distal radius, the distal tibia, and the greater trochanter [40]. Various types of autografts are available as follows.

Cortical bone grafts are best suited for structural defects in which immediate mechanical stability is required for healing [40].

Cancellous bone grafts provide a large surface area leading to a high rate of remodeling and incorporation. Their mechanical strength is limited. Therefore cancellous grafts pose an excellent option for arthrodesis and treatment of nonunions [40].

Corticocancellous bone grafts offer the advantages of both cortical and cancellous bone: an osteoconductive, osteoinductive, and osteogenic properties and immediate structural strength [40]. 

**Figure 2:**
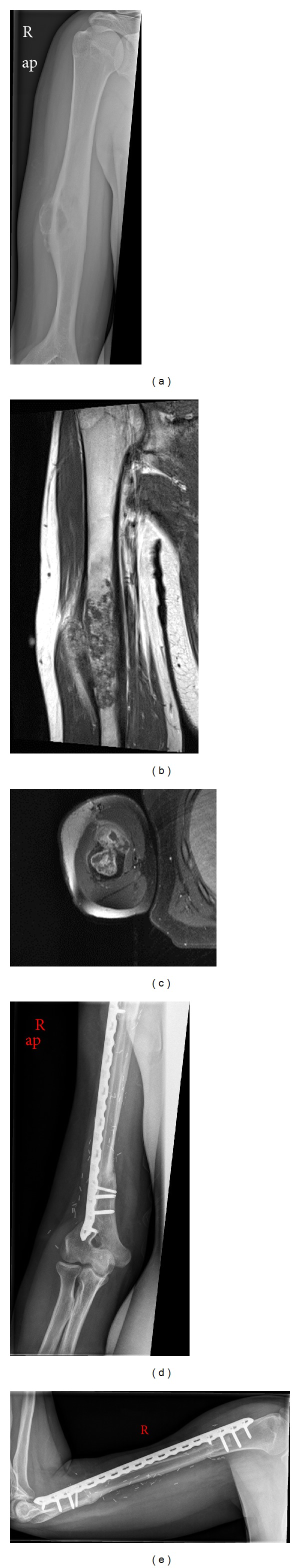
Chondrosarcoma G2 at the proximal humerus shown in the X-ray (a) and MRI ((b), (c)). Nine-month postoperative X-rays showing the reconstruction after wide resection with an autologous free vascularized fibula graft ((d), (e)).

Another option is vascularized bone grafts. These promise the best incorporation and healing due to vascular pedicles. Their use is indicated for large bone defects (>12 cm). The grafts have high osteogenic potential as more than 90% of residual osteocytes survive [40]. Pedicled or free vascularized fibula grafts are among the most commonly used grafts in orthopaedic oncology (Figure 2). They are used in various sites such as humerus, ulna, or radius. Furthermore these grafts pose an option for growth plate reconstruction [29]. 

### 3.6. Complications

Nonunions, fractures, and infections are major complications that are seen frequently in bone autograft transplantations. Union rates are seen in 60% to 90% of vascularized and nonvascularized autografts. In nonvascularized grafts union rate is the lowest with about two thirds. Reconstructions in the upper limbs have significantly higher union rates (90%) compared to lower limbs (70%). Increased union rates can be achieved by the use of additional cancellous bone grafting [29]. An average fracture rate of about 7% is reported in bone autografts use. Fracture rate of transplanted fibulae is high with up to 50%. In cases of fibula graft use solely without any allograft, fracture rates are even higher. In the lower extremity rates are lower and seen in up to a fifth of transplant recipients. Generally, longer grafts are more likely to fracture than shorter grafts [29].

Among the minor complications of bone autografts are donor site pain, superficial nerve injury, hematoma formation, seroma formation, and infection. Early donor site pain occurs quite frequently and can be noticed in up to one third of the patients. Furthermore deep hematomas and infections are reported in about 3%. Among the rare major complications are incisional hernias, sacroiliac joint injury, ureteral injury, gait derangement, and donor site fractures [40].

### 3.7. Plastic Reconstructive Surgery

Soft tissue coverage and reconstruction play an important role in sarcoma surgery as resection often leads to massive soft tissue defects. Standard procedures that are available include skin grafting for superficial defect closure, whereas Vacuum-assisted closure therapy poses an option for temporary defect closure. Since the first microvascular free flaps that were performed in the early 1970s, plastic reconstructive surgery experienced an enormous development. A wide variety of pedicled or free vascularized flaps are available for reconstruction in the whole musculoskeletal system. These include the lateral arm flap, scapula/parascapular flap, radial forearm flap, anterolateral thigh flap, free fibula flap, latissimus dorsi flap, rectus abdominis flap, gracilis flap, free filet flap, the medial femoral condyle periosteal bone flap, or various perforator flaps [41, 42].

## 4. Future

Availability and safety are barriers to the wide use of bone allograft transplantation. However these issues are managed by commercial and noncommercial bone and tissue banks nowadays. So the role of bone allograft transplantation in clinical practice and their outcome will shift into the focus. Minor results and poor function sometimes pose limitations, especially in the use of bone allografts. Furthermore long-term results of transplanted bone allografts show infections, fractures, and nonunions in about 20% of recipients [43]. In general, osteoinduction of allograft is low. Improvement of the incorporation by increasing vascularization, bone remodeling, or osseointegration of bone allograft seems to be one of the major tasks in the future. Various osteoinductive substances have been investigated for bone regeneration [44] that could also improve the incorporation of bone allografts in host tissue. 

Several methods (e.g., growth factors, gene therapy) have been studied that might enhance bone regeneration or repair. Their use might be an interesting option for degenerative or traumatic bone defects. However the use of such methods might not be indicated in oncologic patients as they are seen as potential regulators of cancer cell growth and metastasis [45, 46]. 

Limb lengthening introduced by Ilizarov was a major advancement in orthopaedic practice [47]. Novel techniques were derived using motorized intramedullary nails or magnetically controlled growing rods for osteodistraction. Such nails are available for lengthening of the femur or tibia or correction of spine deformities [48, 49]. Further advancement of such techniques might offer interesting options in the future.

As hand transplantation becomes more frequent, larger transplantations of the upper extremity such as above-elbow arm transplantations are becoming a topic. From the technical point of view such transplantations might be easier to perform as there is just one bone (humerus) and larger vessel compared to further distal sites. However nerve regeneration in larger nerves still poses problems as their structure with multiple fascicles is more complex than that at more peripheral sites [50]. Advancements in this field might revolutionize the management of these procedures [51]. 

As indicated in the introduction salamanders are one of the rare species that keep the potential to regenerate limbs in case of loss throughout their life by nature. So these animals pose a model to study the process of regeneration. A central role in the regenerative capacity has been attributed to fibroblasts that seem to be primarily responsible for the regeneration of limbs in salamanders. Fibroblasts are also present in human wound healing that lead to a scar formation of injured tissue. Therefore much research is performed to study the signals that allow the tissue regeneration instead of the tissue repair. Further understanding of these physiological processes might pose interesting options for further translational research in humans [52].

Concluding we can say that much advancement has been made in the last century so that today there are many highly potential options for good functional biological reconstruction after sarcoma resection that allow patients to regain a high quality of life. 

## References

[1] Karakousis CP (2010). Refinements of surgical technique in soft tissue sarcomas. *Journal of Surgical Oncology*.

[2] Hernroth B, Farahani F, Brunborg G, Dupont S, Dejmek A, Sköld HN (2010). Possibility of mixed progenitor cells in sea star arm regeneration. *Journal of Experimental Zoology B: Molecular and Developmental Evolution*.

[3] Maden M, Turner RN (1978). Supernumerary limbs in the axolotl. *Nature*.

[4] Hutchison C, Pilote M, Roy S (2007). The axolotl limb: a model for bone development, regeneration and fracture healing. *Bone*.

[5] Mavrogenis AF, Coll-Mesa L, Gonzalez-Gaitan M (2011). Criteria and outcome of limb salvage surgery. *Journal of BUON*.

[6] MacEwen W (1881). Observations concerning transplantation of bone: illustrated by a case of inter-human osseous transplantation, whereby over two-thirds of the shaft of a humerus was restored. *Proceedings of the Royal Society (London)*.

[7] MacEwen W (1909). Intrahuman bone grafting and reimplantation of bone. *Annals of Surgery*.

[8] Lexer E (1908). Die Verwendung der freien Knochenplastik nebst Versuchen über Gelenkversteifung und Gelenktransplantation. *Archiv für Klinische Chirurgie*.

[9] Lexer E (1925). Joint transplantation and arthroplasty. *Surg Gynec Obst*.

[10] Carrel A (1912). The preservation of tissues and its applicationin surgery. *The Journal of the American Medical Association*.

[11] Hyatt GW (1950). Fundamentals in the use and preservation of homogenous bone. *United States Armed Forces Medical Journal*.

[12] Hyatt GW, Turner TC, Bassett CA, Pate JW, Sawyer PN (1952). New methods for preserving bone, skin and blood vessels. *Postgraduate medicine*.

[13] Parrish FF (1973). Allograft replacement of all or part of the end of a long bone following excision of a tumor. *Journal of Bone and Joint Surgery A*.

[14] Enneking WF, Burchardt H, Puhl JJ, Piotrowski G (1975). Physical and biological aspects of repair in dog cortical bone transplants. *Journal of Bone and Joint Surgery A*.

[15] Mankin HJ, Fogelson FS, Thrasher AZ, Jaffer F (1976). Massive resection and allograft transplantation in the treatment of malignant bone tumors. *New England Journal of Medicine*.

[16] Burchardt H, Glowczewskie FP, Enneking WF (1983). The effect of adriamycin and methotrexate on the repair of segmental cortical autografts in dogs. *Journal of Bone and Joint Surgery A*.

[17] Hazan EJ, Hornicek FJ, Tomford W, Gebhardt MC, Mankin HJ (2001). The effect of adjuvant chemotherapy on osteoarticular allografts. *Clinical Orthopaedics and Related Research*.

[18] Capanna R, Bufalini C, Campanacci M (1993). A new technique for reconstructions of large metadiaphyseal bone defects: a combined graft (allograft shell plus vascularized fibula). *Orthopedics and Traumatology*.

[19] Peltier LF (1997). Patron saints of medicine. *Clinical Orthopaedics and Related Research*.

[20] Nicoladoni C (1897). Daumenplastik. *Wiener Klinische Wochenschrift*.

[21] Shores JT, Imbriglia JE, Lee WPA (2011). The current state of hand transplantation. *Journal of Hand Surgery*.

[22] Kostiak PE (2000). The evolution of quality systems in human bone banking: the U.S. experience. *Cell and Tissue Banking*.

[23] Boyce T, Edwards J, Scarborough N (1999). Allograft bone: the influence of processing on safety and performance. *Orthopedic Clinics of North America*.

[24] Delloye C, Cornu O, Druez V, Barbier O (2007). Bone allografts. What they can offer and what they cannot. *Journal of Bone and Joint Surgery B*.

[25] Bou Sleiman H, Ritacco LE, Aponte-Tinao L, Muscolo DL, Nolte L-P, Reyes M (2011). Allograft selection for transepiphyseal tumor resection around the knee using three-dimensional surface registration. *Annals of Biomedical Engineering*.

[26] Gebhardt MC, Roth YF, Mankin HJ (1990). Osteoarticular allografts for reconstruction in the proximal part of the humerus after excision of a musculoskeletal tumor. *Journal of Bone and Joint Surgery A*.

[27] Scoccianti G, Campanacci DA, Beltrami G, Caldora P, Capanna R (2010). The use of osteo-articular allografts for reconstruction after resection of the distal radius for tumour. *Journal of Bone and Joint Surgery B*.

[28] Griend RAV (1994). The effect of internal fixation on the healing of large allografts. *Journal of Bone and Joint Surgery A*.

[29] Kunz P, Bernd L (2009). Methods of biological reconstruction for bone sarcoma: indications and limits. *Recent Results in Cancer Research*.

[30] Berrey BH, Lord CF, Gebhardt MC, Mankin HJ (1990). Fractures of allografts. Frequency, treatment, and end-results. *Journal of Bone and Joint Surgery A*.

[31] Mankin HJ, Hornicek FJ, Raskin KA (2005). Infection in massive bone allografts. *Clinical Orthopaedics and Related Research*.

[32] Centers for Disease Control (1988). Transmission of HIV through bone transplantation: case report and public health recommendations. *Morbidity and Mortality Weekly Report*.

[33] Conrad EU, Gretch DR, Obermeyer KR (1995). Transmission of the hepatitis-C virus by tissue transplantation. *Journal of Bone and Joint Surgery A*.

[34] Eggen BM, Nordbo SA (1992). Transmission of HCV by organ transplantation. *The New England Journal of Medicine*.

[35] Tomford WW (1995). Transmission of disease through transplantation of musculoskeletal allografts. *Journal of Bone and Joint Surgery A*.

[36] Buck BE, Malinin TI (1994). Human bone and tissue allografts. Preparation and safety. *Clinical Orthopaedics and Related Research*.

[37] Friedlaender GE, Strong DM, Sell KW (1976). Studies on the antigenicity of bone. I. Freeze dried and deep frozen bone allografts in rabbits. *Journal of Bone and Joint Surgery A*.

[38] Friedlaender GE, Strong DM, Sell KW (1984). Studies on the antigenicity of bone. II. Donor-specific anti-HLA antibodies in human recipients of freeze-dried allografts. *Journal of Bone and Joint Surgery A*.

[39] Stevenson S, Emery SE, Goldberg VM (1996). Factors affecting bone graft incorporation. *Clinical Orthopaedics and Related Research*.

[40] Myeroff C, Archdeacon M (2011). Autogenous bone graft: donor sites and techniques. *Journal of Bone and Joint Surgery A*.

[41] Saint-Cyr M, Wong C, Buchel EW, Colohan S, Pederson WC (2012). Free tissue transfers and replantation. *Plastic and Reconstructive Surgery*.

[42] Choudry UH, Bakri K, Moran SL, Karacor Z, Shin AY (2008). The vascularized medial femoral condyle periosteal bone flap for the treatment of recalcitrant bony nonunions. *Annals of Plastic Surgery*.

[43] Dion N, Sim FH (2002). The use of allografts in musculoskeletal oncology. *Instructional Course Lectures*.

[44] Dimitriou R, Jones E, McGonagle D, Giannoudis PV (2011). Bone regeneration: current concepts and future directions. *BMC Medicine*.

[45] Guo W, Gorlick R, Ladanyi M (1999). Expression of bone morphogenetic proteins and receptors in sarcomas. *Clinical Orthopaedics and Related Research*.

[46] Guise TA, Yin JJ, Taylor SD (1996). Evidence for a causal role of parathyroid hormone-related protein in the pathogenesis of human breast cancer-mediated osteolysis. *Journal of Clinical Investigation*.

[47] Illzarov GA (1971). Osnovnye printsipy chreskostnogo kompressionnogo i distraktsionnogo osteosinteza. *Ortopediia Travmatologiia i Protezirovanie*.

[48] Baumgart R, Betz A, Schweiberer L (1997). A fully implantable motorized intramedullary nail for limb lengthening and bone transport. *Clinical Orthopaedics and Related Research*.

[49] Cheung KM-C, Cheung JP-Y, Samartzis D (2012). Magnetically controlled growing rods for severe spinal curvature in young children: a prospective case series. *The Lancet*.

[50] Lee SK, Wolfe SW (2012). Nerve transfers for the upper extremity: new horizons in nerve reconstruction. *Journal of the American Academy of Orthopaedic Surgeons*.

[51] Jones NF, Schneeberger S (2009). Arm transplantation: prospects and visions. *Transplantation Proceedings*.

[52] Muneoka K, Han M, Gardiner DM (2008). Regrowing human limbs. *Scientific American*.

